# Unraveling an Ultrafast Electron Transport Mechanism in a Photocatalytic “Micromachine” for Their Potential Light Harvesting Applications

**DOI:** 10.3390/mi14050980

**Published:** 2023-04-29

**Authors:** Nivedita Pan, Lopamudra Roy, Md. Nur Hasan, Amrita Banerjee, Ria Ghosh, Meshari A. Alsharif, Basim H. Asghar, Rami J. Obaid, Arpita Chattopadhyay, Ranjan Das, Saleh A. Ahmed, Samir Kumar Pal

**Affiliations:** 1 Department of Chemical and Biological Sciences, S.N. Bose National Centre for Basic Sciences, Salt Lake, Block JD, Sector 3, Kolkata 700106, India; 2 Department of Applied Optics and Photonics, University of Calcutta, 92, Acharya Prafulla Chandra Rd, Machuabazar, Kolkata 700009, India; 3 Department of Physics, Jadavpur University, 188, Raja S.C. Mallick Rd, Kolkata 700032, India; 4 Department of Chemistry, Faculty of Applied Sciences, Umm Al-Qura University, Makkah 21955, Saudi Arabia; 5 Department of Basic Science and Humanities, Techno International New Town, Block-DG 1/2 New Town, Action Area 1, Kolkata 700156, India; 6 Department of Chemistry, West Bengal State University, Barasat, North 24 Parganas, Kolkata 700126, India; 7 Department of Chemistry, Faculty of Science, Assiut University, Assiut 71516, Egypt

**Keywords:** D-π-A organic photosensitizer, wide band gap semiconductor, photocatalysis, ultrafast femtosecond dynamics

## Abstract

Following the seminal discovery of Richard Feynman, several micromachines have been made that are capable of several applications, such as solar energy harvesting, remediation of environmental pollution, etc. Here we have synthesized a nanohybrid combining TiO_2_ nanoparticle and light harvesting robust organic molecule RK1 (2-cyano-3-(4-(7-(5-(4-(diphenylamino)phenyl)-4-octylthiophen-2-yl)benzo[c][1,2,5] thiadiazol-4-yl)phenyl) acrylic acid) as a model micromachine having solar light harvesting ability potential for application in photocatalysis, preparation of solar active devices, etc. Detailed structural characterization, including High Resolution Transmission Electronic Microscopy (HRTEM) and Fourier-transform infrared spectroscopy (FTIR), has been performed on the nanohybrid. We have studied the excited-state ultrafast dynamics of the efficient push-pull dye RK1 in solution, on mesoporous semiconductor nanoparticles, and in insulator nanoparticles by streak camera (resolution of the order of 500 fs). The dynamics of such photosensitizers in polar solvents have been reported, and it has been observed that completely different dynamics occur when they are attached to the surface of the semiconductor/insulator nanosurface. A femtosecond-resolved fast electron transfer has been reported when photosensitizer RK1 has been attached to the surface of the semiconductor nanoparticle, which in turn plays a crucial role in the development of an efficient light harvesting material. The generation of reactive oxygen species as a result of femtosecond-resolved photoinduced electron injection in the aqueous medium is also investigated in order to explore the possibility of redox-active micromachines, which are found to be crucial for efficient and enhanced photocatalysis.

## 1. Introduction

The 21st century has witnessed significant challenges arising from the growing demand for energy and the resulting environmental impacts [1,2,3]. Solar radiation is deemed the primary green and renewable energy source among others, owing to its consistent and abundant existence on the earth’s surface [4,5]. The efficient harvesting of solar energy into chemical and electrical energy can solve the energy crisis as well as the alarming environmental pollution the earth is facing today [6,7]. Numerous studies have reported the generation of reactive oxygen species (ROS) [8] from the water molecules or from the dissolved oxygen in water via various nanohybrids on the absorption of light as a technique for conversion of solar energy to chemical energy [9,10]. The generated ROS has the potential to exert antimicrobial action and can degrade both organic and inorganic pollutants present in water [11,12,13]. Photocatalysis is a well-known technique that utilizes the ROS generated from recyclable nanomaterials and degrades organic pollutants by producing oxidizing free radicals [14].

As a result, the development of novel nanomaterials with efficient photocatalytic activity, high stability, and a significantly good absorption window in the solar spectrum has been regarded as a critical prerequisite for combating environmental pollution via solar energy conversion [15]. Among many, TiO_2_ can be called the best semiconductor photocatalyst due to its unique features. To improve its photocatalytic properties, TiO_2_ can be conjugated into a range of materials, including metals, nonmetals, and other photocatalysts, to form different types of hybrids. The photoresponse can be improved in the visible region by sensitizing mesoporous wide-bandgap semiconductor photocatalysts (TiO_2_ and ZnO) with sensitizers and tuning the absorption window accordingly. This method captures attention because of its promising response towards cheaper and more environment friendly approaches, where the choice of sensitizers has become equally important. However, the poor photocatalytic efficiency, dye leaching, and degradation under solar irradiation remain concerns. Thus, the choice of the sensitizer material and its constructional arrangement play a crucial role. Along with it, the electron transfer phenomena at the TiO_2_/sensitizer interface have been found to play a key role in efficient light-harvesting materials. Here, ultrafast charge injection of the order of femtosecond time scales can play a predominant role [16] which is still a less explored area.

Organic photosensitizers with a delocalized system of electrons, which are end-capped with electron acceptors (A) and donors (D) due to their simple synthesis techniques, robust stability, high molar extinction coefficients, and exceptionally tunable absorbances, have been thoroughly investigated for the last four decades [17,18]. The widespread applications of organic photosensitizers have been explored across various branches of modern research, including solar light harvesting, due to their femtosecond-resolved ultrafast response [16,17]. In this direction, the dynamics of such D-π-A sensitizers are sometimes crucial to understand in their pure state and when attached to a semiconducting/insulating substrate. Additionally, because of the presence of ultrafast spectral/electronic relaxation, femtosecond-resolved ultrafast tools are required for their dynamic understanding.

In this work, we have used RK1, which is a commercially available triphenylamine-based sensitizer with a D-π-A structure, and a femtosecond-resolved streak camera setup for dynamical understanding. RK1 is a highly stable chromophore under solar light irradiation, and RK1-sensitized TiO_2_-based Dye-Sensitized Solar Cells (DSSCs) have been reported with a power conversion efficiency of around 10% [19,20]. D-π-A structural photosensitizers having D-A interaction account for even more interesting optoelectronic behavior, such as the formation of new lower energy molecular orbitals (MO) termed intramolecular charge transfer states (ICT) [21]. ICT is responsible for the polar nature of such sensitizers, and thereby we have observed such behaviors of RK1 in the polar solvent, mesoporous RK1-TiO_2_ nanohybrid, and on RK1-Al_2_O_3_ nanohybrid using steady-state and femtosecond-resolved fluorescence spectroscopy-based tools. It was found that compared to pure solvent, the spectral and time-resolved responses of such photosensitizers when grafted on semiconducting nanosurfaces get completely changed, which is again responsible for ROS generation and their application in photocatalysis. Here we have shown that due to the presence of ultrafast electron transfer from RK1 TiO_2_, besides the use of organic photosensitizer RK1 as a high-efficiency material in DSSCs, it is an important environment-friendly material as a photocatalyst as well.

## 2. Models and Methods

### 2.1. Reagents

Titanium (IV) oxide nanoparticles (TiO_2_, ~21 nm) and Acridine orange (AO) were purchased from Sigma-Aldrich. 2-Cyano-3-(4-(7-(5-(4-(diphenylamino)phenyl)-4-octylthiophen-2-yl)benzo[c][1,2,5] thiadiazol-4-yl)phenyl) acrylic acid (RK1) was purchased from Solaronix. Dichlorofluorescein diacetate (DCFHDA) and ethanol (solvents) were purchased from Merck. All the reagents were of analytical grade and used without further purification. The Milli-Q water used was from Millipore.

### 2.2. Synthesis of Hybrid Materials

The RK1-TiO_2_ nanohybrid was synthesized by adding 100 mg of TiO_2_ NPs into 10 mL of 0.3 mM solution of RK1 prepared in ethanol. This mixture was kept under constant magnetic stirring at room temperature for 6 h. The solution was then successively washed with ethanol several times by centrifuging it for 10 min to discard the loosely bound or unbound dyes from the surface of the TiO_2_ NPs. Finally, the prepared nanohybrid was dried at 60 °C. The same procedure was followed to synthesize RK1-Al_2_O_3_ nanohybrids; the only difference was that Al_2_O_3_ was used in place of TiO_2_.

### 2.3. Methods of Characterization

A Shimadzu UV-2600 spectrophotometer was used to record all the absorption spectra. All the steady-state emission and excitation spectra were recorded using a Jobin Yvon Fluorolog fluorimeter. A Panalytical XPERTPRO diffractometer equipped with Cu Kα radiation (at 40 mA and 40 kV) with a scanning speed of 0.02 s^−1^ and 2θ ranges from 20° to 80° was used to perform X-ray powder diffraction (XRD) measurements of the prepared nanoparticle and nanohybrid powder at room temperature. The geometry of the synthesized nanohybrid was evaluated by transmission electron microscopy (TEM) studies using an FEI (Technai S-Twin) instrument with an acceleration voltage of 200 kV. Samples for the TEM measurement were prepared by drop casting the nanohybrid solution on a carbon-coated circular (3 mm diameter) copper grid. Fourier transform infrared spectroscopy (FTIR) of the powder samples was performed with the attenuated total reflectance (ATR) method using a Vertex 70 V instrument (Bruker, Germany). Thermogravimetry (TG) (the variation of weight with respect to temperature during the phase transition of a material) for the samples was measured using a PerkinElmer TGA-50H instrument under a nitrogen atmosphere, and the samples were heated from 30 to 600 °C at a rate of 10 °C/min.

### 2.4. Femto-Second-Resolved Fluorescence Measurements Using Streak Camera Images

Femtosecond-resolved fluorescence lifetime decay measurements were carried out by capturing the streak camera images in a custom-made steak camera-based setup. The customized streak camera system consists of 3.5 ps FWHM. Laser source (Mai Tai HP 1040S from Spectra Physics, Mode-Locked Ti:sapphire Laser, 2.5 W, repetition rate 80 MHz, working temperature 20–25 °C), sample compartment, monochromator (SpectraPro HRS-300, scan range 0–1500 nm, 300 mm triple grating, grating change repeatability 0.2 nm), streak sweep unit, along with a Charge Coupled Device (CCD) camera (Optronics OPTOSCOPE SC-10). The fluorescence light from the exit slit of the monochromator is guided to the photocathode of the streak camera to produce a horizontal linear spectral image [22]. The produced photoelectrons are then accelerated further by the electrical field and biased in the vertical direction under a variable electrical field through a streak sweep unit. These photoelectrons impinge on the multichannel plate (MCP) inside the streak camera and make multiple electrons that give a single spot on the target phosphor screen [23]. The field scan and the laser pulse flashing are synchronized. These spots are then recorded by CCD, and the corresponding pixel information is transferred to a computer for further data processing. The streak camera produces 2-dimensional (2D) spectrum-time images (290 ps time along the x-axis for a 15 ps/mm sweep rate span and a 150 nm wavelength span along the y-axis). A streak image helps detect both wavelength and time from the same laser pulse shot. This experiment effectively captures nearly all photons of various wavelengths and times without producing artifacts from spectrum or decay changes during lengthy measurements. The decay of the fluorescence intensity in the excited state could be represented as It=I0e−tτ, where *I*(*t*) is the recorded intensity, I_0_ is the initial intensity, and τ is the fluorescence lifetime. The above expression gets changed for multiexponential fluorescence decays. Here, to fit the decay, multiple decay components were used, as the decays were multiexponential in nature. Detailed fitting procedures are explained in “Principles of Fluorescence Spectroscopy” by Lakowicz [24]. Micromath Scientist software, which employs deconvolution via the iterative reconvolution technique based on a nonlinear least-squares process, was used to fit fluorescence transients.

### 2.5. Dichlorofluorescein (DCFH) Test for ROS Generation

A DCFH solution was prepared by mixing 0.5 mL of 1.0 mM DCFH-DA in methanol with 2.0 mL of 0.01 N aqueous NaOH. After mixing, this solution was kept at room temperature for 30 min. The pH of the mixture was then adjusted to 7.4 by adding 10 mL of 25 mM NaH_2_PO_4,_ as reported earlier [25,26]. The DCFH solution was stored on ice in dark conditions. In this assay, excitation with a 488 nm light beam leads DCFH to get oxidized and produce fluorescent DCF, which reflects the generation of ROS. Using steady-state fluorescence emission after excitation at a frequency of 488 nm, the fluorescence intensity of DCF at about 520 nm can be measured. Fresh DCFH was introduced to the system every hour during the recyclability assay, which was carried out three times in a row. A warm white light (Havlock, 12 W) of 42,800 lux was used to illuminate the samples.

### 2.6. Methods for Photocatalysis Test

Under visible light illumination, the photocatalytic activity of the nanohybrids was tested to detect the photo-decomposition of acridine orange (AO), a model pollutant with a nitrogen-containing heterocyclic structure in aqueous media. The degradation of AO under light illumination (initial concentration C = 0.5 × 10^−4^ M) was performed in a 1 cm optical path length quartz cuvette containing 3 mL of solution with a 1 g L^−1^ concentration of each nanohybrid. The solution was illuminated with a warm white light (Havlock, 12 W) of 42,800 Lux. The degradation percentage (*%DE*) of AO in 1 h was calculated by using the equation $\%\;DE=\frac{I_{0}-I}{I_{0}}\times 100$, where *I*_0_ represents the initial absorption intensity of AO at its maximum absorbance peak, λ_max_ = 491 nm and I represents the absorption intensity after getting irradiated for 1 h.

## 3. Result and Discussion

### 3.1. Optical and Structural Characterization

**Figure 1:**
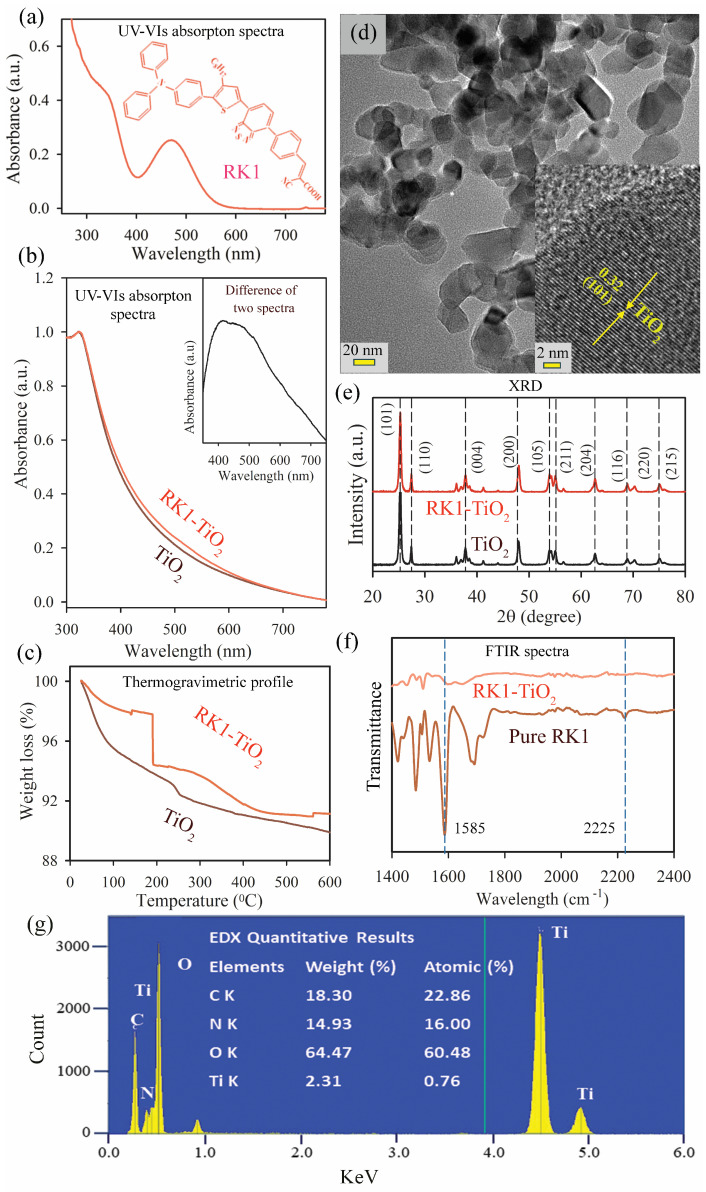
(**a**) UV-vis absorption spectrum of RK1 in ethanol solvent, inset shows the molecular structure of RK1. (**b**) Normalized UV-vis absorbance spectra of TiO_2_ (brown) and RK1-TiO_2_ (red), inset shows the differential absorbance spectrum of TiO_2_ and RK1-TiO_2_. (**c**) Corresponding Thermogravimetric profile of TiO_2_ (brown) and RK1-TiO_2_ (red) monitored under N_2_ flow. (**d**) TEM image of the RK1-TiO_2_ nanohybrid, inset shows the HRTEM image of the RK1-TiO_2_ nanohybrid (**e**) XRD patterns of TiO_2_ (black) and RK1-TiO_2_ (red) nanohybrid. (**f**) FTIR spectra of powdered pure RK1 (brown) and RK1-TiO_2_ (red) nanohybrid. (**g**) The Energy dispersive X-ray spectrum of RK1-TiO_2_ nanohybrid, and the corresponding elemental quantitative results.

**Figure 2:**
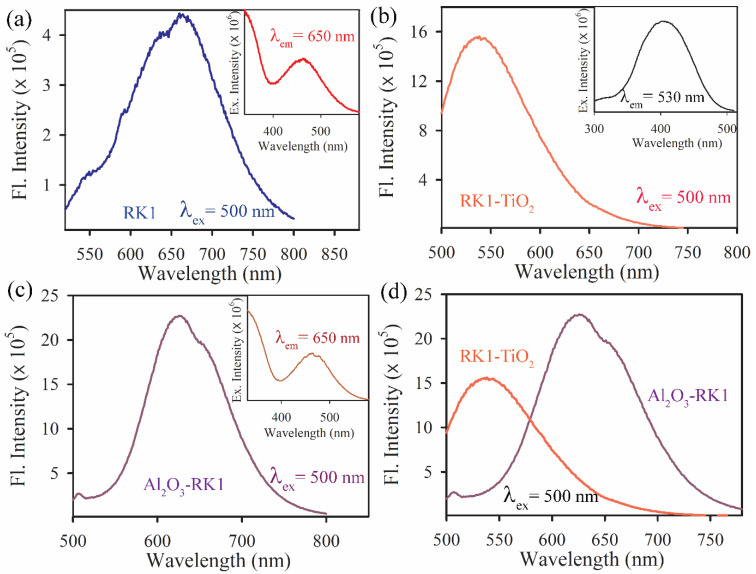
The steady-state fluorescence emission spectra of (**a**) RK1, (**b**) RK1-TiO_2_ nanohybrid, and (**c**) Al_2_O_3_-RK1 nanohybrid at an excitation wavelength of 500 nm. The insets of each show their respective excitation spectra. (**d**) Comparative emission spectra of Al_2_O_3_-RK1, and RK1-TiO_2_ nanohybrids.

RK1 is a donor-bridge acceptor-type photosensitizer with strong absorption in the visible region. It is easily soluble in most polar solvents, such as ethanol, ACN, THF, etc., and in some non-polar solvents. We have used ethanol as a solvent for the preparation of nanohybrids in our study. The optical absorption properties of the dye have been measured using UV-vis spectroscopy and are shown in Figure 1a. The interaction between the donor and acceptor groups, known as the internal charge transfer (ICT) state, results in the absorption peaks of RK1 at 346 nm in the UV region and 470 nm in the visible region. The onset of Figure 1a shows the structure of RK1, which has a triphenylamine electron-donating group and cyanoacrylic acid as an electron-withdrawing group. Sensitization of the TiO_2_ surface has been a widely accepted method for achieving a broader absorption window in applications where electron hole pair separation under light irradiation is the basic phenomenon. Among them, photocatalysis is the most common technique to deal with environmental pollution monitoring [27]. The use of this dye was first reported in DSSSc [19] where it was used to sensitize the TiO_2_ surface. In the present study, we have prepared the nanohybrid with RK1-sensitized TiO_2_. The absorption of TiO_2_ and RK1-TiO_2_ has been shown in Figure 1b. The absorption of TiO_2_ is in the UV region, with a peak maximum of 325 nm. For the RK1-TiO_2_ nanohybrid, the attachment of RK1 is distinguishable from 400 to 700 nm. The difference between the two spectra has also been plotted and shown in the inset of Figure 2, where a significant contribution of RK1 appears. Thermogravimetric analysis (TGA) has been performed to determine a material’s thermal stability before and after RK1 loading on the surface of TiO_2_. Figure 1c depicts the TGA profiles of TiO_2_ (black) and TiO_2_-RK1 nanohybrid (red). It shows that the nanohybrid is more stable than pristine TiO_2_ nanoparticles due to the enfolding of RK1 in the pores of TiO_2_ [28]. The dye loading capability is calculated from the TGA plot in temperature ranges from 180 °C to 450 °C, which have found to be 49%. Such efficient dye loading is beneficial for environmental applications. TEM and high-resolution TEM (HRTEM) have been used to examine the structural morphology of the nanohybrid. The nanoparticles of the hybrid have an average diameter of around 30 nm (Figure 1d), which is consistent with TiO_2_ nanoparticles [20]. Inset shows the HRTEM image of the nanohybrid (inset of Figure 1d), where the fringe width of 0.32 nm corresponds to TiO_2_ rutile 101 phases. Phase structural characterization of the TiO_2_ and nanohybrid has been determined by powder X-ray diffraction. Figure 1e demonstrates the diffraction patterns of TiO_2_ (black) and the synthesized RK1-TiO_2_ nanohybrid (red). For two samples, the characteristic diffraction peaks at around 27°, 36°, 55°, and 25°, 48°, corresponding to planes (110), (004), (211), and (101), (200), respectively, refer to the presence of the mixed rutile and anatase phases of TiO_2_ [29]. It has been shown from X-ray diffraction and TEM analysis that there is no change in morphology or phase composition after hybrid formation. Furthermore, the mode of attachment of RK1 to the TiO_2_ surface has been investigated with the help of FTIR spectra. As shown in Figure 1f, the FTIR spectra of the pure RK1 and RK1-TiO_2_ hybrids corroborate the attachment of the dye on the TiO_2_ surface through the joining groups of cyanoacrylic acid, hydroxyl group, and cyano group. A clear decrement of peak intensity along with a shift of the vibrational modes, 1698 cm^−1^ C=O vibration of COOH [30] of the nanohybrid compared to RK1 in its pure state, is observed, giving evidence of bridged bidentate binding [31] whereas the peak corresponding to 1585 cm^−1^ of COO^−^ [30] and 2225 cm^−1^ of C≡N is almost suppressed in the hybrid system, indicating the presence of a COO/CN (A2) binding mode [31]. These spectral changes in the anchoring groups confirm the formation of the RK1-TiO_2_ hybrid. In addition to it, we have also performed energy dispersive X-ray (EDX) spectral analysis for elemental quantification in the prepared hybrid nanomaterial, as shown in Figure 1g. The presence of carbon (C), nitrogen (N), titanium (Ti), and oxygen (O) is due to the organic ligand RK1.

For applications of such nanohybrids towards efficient photocatalysis, the steady state and the femtosecond resolved fluorescence transients of the sensitizer and its nanohybrid with TiO_2_ were performed for understanding the photoinduced chemical and physical phenomena associated with the processes of photon harvesting and exciton separation. As shown in Figure 2a, RK1 in ethanol has an emission maximum at 650 nm when excited at 500 nm, corresponding to its absorption maximum, whereas the excitation spectrum (inset of Figure 2a) resembles the absorption spectrum. Whereas, as shown in Figure 2b, RK1-TiO_2_ nanohybrid, when dispersed in ethanol solvent, indicates a shift in emission spectrum to 550 nm with an increase in fluorescence intensity, with excitation spectra having a peak maximum at around 400 nm (inset of Figure 2a). This blue shift in emission spectra suggests the formation of a higher energy intermediate state with an absorption maximum at around 400 nm, as confirmed by the excitation spectra. We have further grafted RK1 on the surface of an insulator so that a comparison could be made due to the electron/charge transfer possibility from the dye to the semiconductor. RK1, after forming a nanohybrid with Al_2_O_3_, shows similar behavior, i.e., the emission intensity has increased many times compared to the dye in pure solvent. However, no such shift in the emission maximum of Al_2_O_3_-RK1 has been found. As depicted in Figure 2c, the maximum of fluorescence (FL) at around 650 nm and the absorption maximum at 480 nm resemble excitation spectra. The reduction in fluorescence intensity of RK1 in a polar solvent (ethanol) can be attributed to electronic relaxation from the initially excited Frank condom state (FC) toward an excited state with a more favorable charge transfer (CT) option. This state, mainly formed in polar solvents, has been described in the literature [32] for push-pull dye and is less conjugated in nature, resulting in a decrease in fluorescence intensity. Additionally, the FL quenching in the presence of TiO_2_ due to electron transfer from the dye to the semiconductor substrate compared to that of an insulator like Al_2_O_3_ has been shown in Figure 2d.

**Figure 3:**
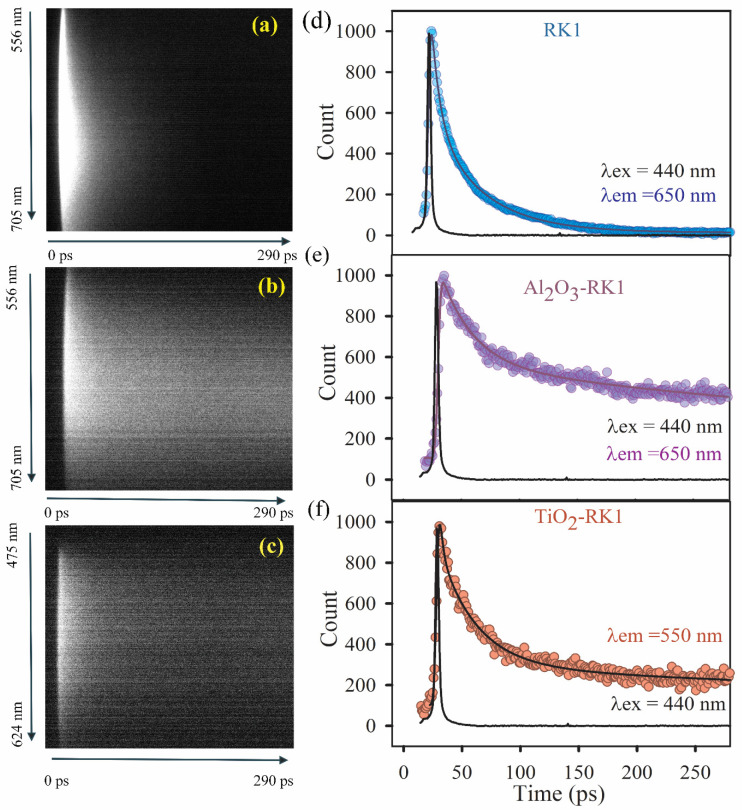
Femtosecond resolved time-wavelength 2D fluorescence decay images of (**a**) RK1 at 650 nm, (**b**) Al_2_O_3_-RK1 nanohybrid at 650 nm, and (**c**) TiO_2_ -RK1 nanohybrid, at 550 nm in ethanol solvent. The excitation laser was 440 nm (with a 3.5 ps pulse width). [Measurements were conducted in the 290 ns time window. The vertical and horizontal axes represent fluorescence emission wavelength, and time respectively.] Corresponding femtosecond resolved fluorescence decay transients of (**d**) RK1 at 650 nm, (**e**) Al_2_O_3_-RK1 nanohybrid at 650 nm, and (**f**) TiO_2_-RK1 nanohybrid at 550 nm.

Such push-pull dyes involve femtosecond-induced chemical and physical properties and have been analyzed through the customized streak camera setup. The photoexcited decays of charge carriers have been analyzed, and the fitted time parameters are summarized in Table 1. The 2D spectrum-time image of RK1 in ethanol is represented in Figure 3a when excited with the second harmonic (440 nm, 3.5 ps FWHM) of the femtosecond laser pulse, consisting of a femtosecond-resolved decay having maximum spread at around 650 nm. To get the component value information of transients decay at FL peak maximum, a streak consisting of 5 nm width along the y-axis centered at 650 nm has been chosen, and the corresponding FL decay transient (Figure 3d) depicts two decay components of the order of 7.2 ps having a 54% contribution and 42.8 ps having a 46% contribution. The faster 7.2 ps component may be attributed to the electronic relaxation from the FC state to the CT state, and the later 42.8 ps resembles the ground state relaxation of the CT state. The dye, when attached to the Al_2_O_3_ surface, shows a completely different behavior. The 2D spectrum-time image (Figure 3b) of RK1-Al_2_O_3_ depicts two different transient decay components: one is of the order of tens of picosecond while the other is of the order of hundreds of picoseconds. The decay components are 28 ps (51% contribution) and 580 ps (49% contribution). The 28 ps component can be attributed to a possible injection from the dye excited state to the Al_2_O_3_ defect/trap states or spectral relaxation as an electronic transition mentioned above from the FC state to the more polar CT state. However, the relaxation here is not as fast (28 ps) as in the case of dye in solvent (7 ps) because of nanohybrid formation, and the ground state relaxation from the CT state happens in the order of 580 ps. From the streak spectrum-time image (Figure 3c) of RK1-TiO_2_, it has been observed that along with these components, a component of an even faster time scale, i.e., 10 ps with a 40% contribution exists (Figure 3f). This faster femtosecond timescale component is only due to the electron injection from the excited-state photosensitizer to the semiconducting TiO_2_ substrate. The decay has been captured in this case at a 550 nm cantered streak window, which corresponds to its FL maximum.

### 3.2. Photocatalytic Performance

**Figure 4:**
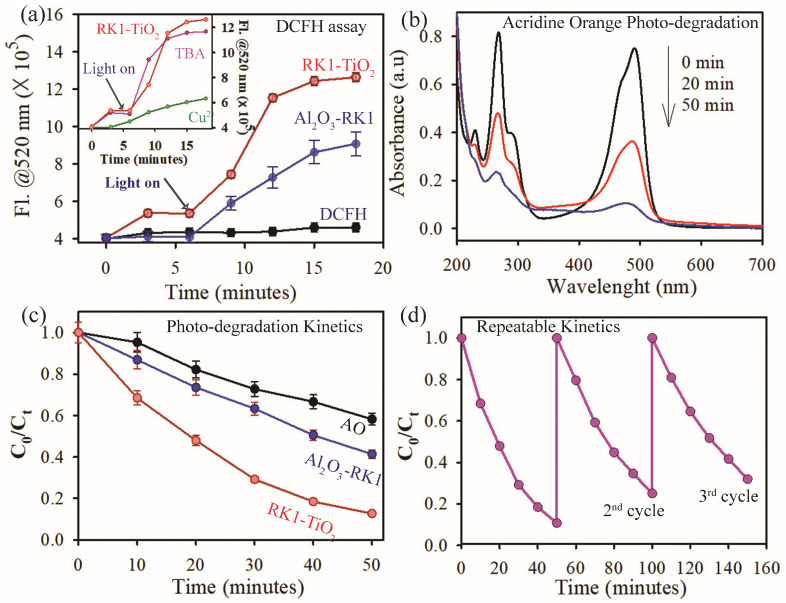
(**a**) DCFH oxidation with respect to time with the addition of RK1-TiO_2_ nanohybrid (brown) Al_2_O_3_-RK1 (Blue) and DCFH control (black) under white light irradiation monitored at 520 nm. Inset shows the RK1-TiO_2_ ROS activity in the presence of radical scavengers; Cu^2+^ (green), and tert butyl alcohol (magenta), without any scavenger (red). (**b**) Photocatalytic degradation of acridine orange (AO) by RK1-TiO_2_ nanohybrid. (**c**) Comparison of catalytic activity of RK1-TiO_2_ with Al_2_O_3_-RK1 and AO itself at the absorption peak maximum of AO. (**d**) Recyclability of the catalyst RK1-TiO_2_ nanohybrid.

In order to investigate the effect of such fast electron injection from the excited state of D-π-A visible absorbing chromophore to a wide band gap semiconductor, ROS generation and ROS-mediated photocatalysis towards pollutant degradation under white light irradiation have been monitored. The DCFH assay is a well-known technique to monitor ROS generation in water media. RK1-TiO_2_ nanohybrid powder has been used for the photocatalytic measurements under study. From Figure 4a, it is evident that the increase in DCF emission at 520 nm upon oxidation of DCFH through ROS generation, which increases with photoexcitation of the nanohybrid under white light, Due to the sensitization of TiO_2_ with RK1, a broader absorbance of RK1-TiO_2_ has been observed, which thereby shows about three times higher ROS generation in water media compared to the control Al_2_O_3_-RK1. The higher ROS generation in the presence of TiO_2_ compared to that of Al_2_O_3_ signifies the role of ultrafast electron transfer from the conduction band of RK1 to semiconductor TiO_2_. Furthermore, this generation of reactive oxygen species, such as •OH and O_2_^•−^, has been utilized for photocatalytic pollutant degradation. To explore the type of ROS generated, we have performed ROS generation experiments in the presence of excess radical scavengers, and the findings are shown in the inset of Figure 4a. In the presence of Cu^2+^, a well-known electron scavenger, the ROS generation ability of RK1-TiO_2_ is drastically reduced. It implies the presence of O_2_^•−^ in the medium, and due to it, Cu^2+^ gets converted into Cu^+^ while accepting the electron from the medium. Whereas, in the presence of tertiary butyl alcohol (TBA), which is a scavenger of •OH, no such change in ROS generation has been observed, which clearly indicates that among other reactive species, the negatively charged O_2_^•−^ plays the most crucial role in ROS generation. We have here used AO as a model pollutant that would be degraded by recyclable TiO_2_-RK1 nanohybrids. For the photocatalysis experiment, the nanohybrid and the AO solution were priorly shaken well and kept under dark conditions for 1 h so that they could attain equilibrium after limited adsorption of AO on the surface of the nanohybrid. The measure of photocatalysis is the amount of degradation of AO itself in the presence of a photocatalyst-nanohybrid with light irradiation. Figure 4b shows the photocatalytic degradation profile of AO under white light irradiation; the discoloration of the AO solution and the absorption shift of AO indicates the formation of non-harmful proflavine [33]. To confirm the enhanced AO degradation due to the femtosecond timescale faster electron injection from photoexcited RK1 to TiO_2_, control AO degradation experiments were performed with the RK1-Al_2_O_3_ nanohybrid and AO itself. From Figure 4c, RK1-TiO_2_ shows maximal photocatalytic activity as observed for the duration of 50 min. Recyclability of the used photocatalyst is one of the important parameters in designing such materials; the intact nature of the FTIR vibrational mode indicates the integrity of the nanohybrid after light irradiation. We have found from Figure 4d that our photocatalyst is almost 40% active even after three cycles. The reason behind the decrease in photocatalytic activity after three cycles may be attributed to the agglomeration of reaction products on the surface of the photocatalytic material. These reaction products can block the active sites on the surface of the material, reducing the surface area available for reaction. In addition, the photocatalytic material may also experience surface damage over time due to repeated exposure to light and the generation of reactive species.

**Scheme 1:**
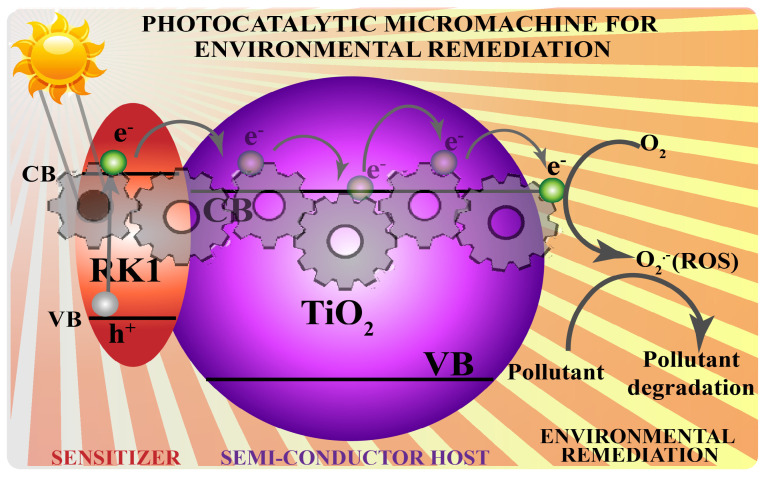
The RK1-TiO_2_ is a photocatalytic micromachine for environmental remediation.

It can be proven from here that electron injection from dye to the semiconductor surface plays a crucial role in ROS-mediated photocatalysis. A schematic representation (Scheme 1) has been shown for more clarification regarding electron transfer and ROS generation.

## 4. Conclusions

Our present work on a model D-π-A sensitizer, RK1, gives insights into its excited state dynamical behavior for potential application in photocatalysis as a light harvesting photosensitizer when attached to the surface of TiO_2_ nanoparticles. The fact to be noticed here is that an ultrafast femtosecond-resolved electron injection component of 10 ps has been observed from the excited state of RK1 to TiO_2_, which here plays a crucial role in enhanced photocatalytic activity through ROS generation. We have used a femtosecond laser pulse of 3.5 ps to excite the samples in the visible region and monitored the excited state dynamics in a femtosecond-resolved streak camera setup. Our work will open up further directions toward robust, stable, and high-efficiency photocatalysis using D-π-A type sensitizer nanohybrids.

## Figures and Tables

**Table 1:** Dynamics of femtosecond resolved fluorescence transients of RK1, Al_2_O_3_-RK1, and TiO_2_-RK1.

Sample	Excitation Wavelength (nm)	Emission Wavelength (nm)	$\tau_{1}\;$ (ps)	$\tau_{2}\;$ (ps)	$\tau_{3}\;$ (ps)	$\tau_{avg}\;$ (ps)
RK1	440	650	7.24 (54%)	42.8 (46%)	_	23.56
Al_2_O_3_-RK1	440	650	28.32 (51%)	579.4 (49%)	_	297.76
TiO_2_-RK1	440	550	10.00 (40%)	31.57 (45%)	523.26 (15%)	95.92

## Data Availability

Data will be available upon request from the corresponding authors.
